# Anti-Inflammatory Effects of TRAF-Interacting Protein in Rheumatoid Arthritis Fibroblast-Like Synoviocytes

**DOI:** 10.1155/2016/3906108

**Published:** 2016-10-26

**Authors:** Qing-Zhu Kong, Li-Tao Guo, Jia-Ning Yang, Yan-Fei Wang, Jing-Xin Zhao, Su-Hong Kong, Meng Zhang, Shi Yan, Yu Jin

**Affiliations:** ^1^Department of Orthopedic Trauma, Affiliated Hospital of Chengde Medical University, Chengde, Hebei 06700, China; ^2^Department of Ophthalmology, Affiliated Hospital of Chengde Medical University, Chengde, Hebei 06700, China; ^3^Department of Spinal Surgery, Affiliated Hospital of Chengde Medical University, Chengde, Hebei 06700, China; ^4^Department of Emergency, Affiliated Hospital of Chengde Medical University, Chengde, Hebei 06700, China; ^5^Department of Gynaecology and Obstetrics, Maternal and Child Health Care Hospital of Longhua County, Chengde, Hebei 068150, China

## Abstract

Rheumatoid arthritis (RA) is a chronic systemic inflammatory disease characterized by inflammatory cell infiltration, synovial inflammation, and cartilage destruction. Proliferative fibroblast-like synoviocytes (FLS) play crucial roles in both propagation of inflammation and joint damage because of their production of great amount of proinflammatory cytokines and proteolytic enzymes. In this study, we investigate the role of TRAF-interacting protein (TRIP) in regulating inflammatory process in RA-FLS. TRIP expression was attenuated in RA-FLS compared with osteoarthritis- (OA-) FLS. Overexpression of TRIP significantly inhibited the activation of NF-*κ*B signaling and decreased the production of proinflammatory cytokines and matrix metalloproteinases (MMPs) in TNF*α*-stimulated RA-FLS. Furthermore, TRIP was found to interact with transforming growth factor *β*-activated kinase 1 (TAK1) and promoting K48-linked polyubiquitination of TAK1 in RA-FLS. Our results demonstrate that TRIP has anti-inflammatory effects on RA-FLS and suggest TRIP as a potential therapeutic target for human RA.

## 1. Introduction

Rheumatoid arthritis (RA) is a kind of chronic systemic autoimmune disease, accompanied by the destruction of cartilage and bone [1]. Fibroblast-like synoviocytes (FLS) in synovial tissues have been shown to play crucial roles in the pathogenesis of RA, such as initiation of inflammatory responses and inflammation-associated cartilage damage [2]. Despite the rapid progress of modern medicine, there is still a lack of effective treatment for rheumatoid arthritis. Therefore, it is of great significance to explore the precise etiology and underlying mechanisms of RA and identify novel therapeutic targets [3].

During the progression of RA, constant inflammatory responses occur in the synovial membrane, accompanied by the increased FLS cell number, and changed proliferative and apoptotic properties of FLS. FLS together with other immune cells, including macrophages, dendritic cells, and lymphocytes, could disrupt immune homeostasis and create an inflammatory environment in the synovium, which contributes to cartilage and joint destruction [4, 5]. In addition, FLS can promote various processes in RA by secreting different types of inflammatory cytokines, such as IL-6, IL-8, IL-1*β*, TNF*α*, and MCP-1, and matrix metalloproteinases (MMPs), such as MMP-1 and MMP-13, which has been shown to be closely related to the activation of cellular mitogen-activated protein kinase (MAPK) and nuclear factor-kappaB (NF-*κ*B) signaling pathways [6–9]. Because of the pivotal roles of FLS in the pathogenesis of RA, recent studies have reported that it could be potential approaches for the treatment of RA, mainly through the modulation of activities of various essential adaptors and nuclear transcriptional factors in the inflammation relevant signaling pathways in FLS [10–14].

The tumor necrosis factor (TNF) receptor-associated factor- (TRAF-) interacting protein (TRIP/TRAIP) belongs to the RING-type E3 ubiquitin ligase family, and it is composed of the N-terminal RING-finger motif that is followed by coiled-coil and leucine zipper-like domains that bind TRAF-family proteins [15] and C-terminal domain which could interact with CYLD and repress the activation of NF-*κ*B signaling in a RING domain independent manner [16]. In addition, TRIP was reported as a regulator in IFN-*β* signaling by mediating ubiquitination and degradation of TBK1 through the N-terminal RING domain [17]. TRIP could also interact with the protein tyrosine kinase Syk in breast epithelial cells, and Syk enhances the activation of NF-*κ*B by TNF*α* and this is antagonized by TRIP [18]. Overexpression of TRIP has been reported in basal cell carcinomas [19], breast cancer [18, 20], and TRIP mRNA and protein expression correlated with a peak in the G2/M phase suggesting that TRIP has physiological function in G2/M [21]. But the function of TRIP in RA and the underlying mechanism still remains unknown.

In this report we showed that TRIP expression was attenuated in RA-FLS compared with OA-FLS, and overexpression of TRIP significantly inhibited the activation of NF-*κ*B signaling and decreased the production of proinflammatory cytokines and MMPs in TNF*α*-stimulated RA-FLS. We also found that TRIP could interact with TAK1 and promoted K48-ubiquitination and degradation of TAK1 in RA-FLS. In conclusion, we revealed an anti-inflammatory role of TRIP in RA in current study.

## 2. Material and Methods

### 2.1. Patients

Ten Chinese OA patients and Ten Chinese RA patients were enrolled from the Department of Rheumatology of Affiliated Hospital of Chengde Medical University. RA was diagnosed according to 2010 ACR/European League against Rheumatism (EULAR) classification criteria for RA and the evaluation of 28-joint disease activity score (DAS28) of the RA patients was all over 2.6 [22]. The study protocol was approved by the Ethics Committee of Our Hospital (IRB-S1126) and all patients gave written informed consent.

### 2.2. Synovial Tissue Collection and FLS Culture

The collection of synovial samples by needle arthroscopy from RA and OA patients was performed as previously described [23, 24]. FLS were isolated from the synovial tissues by modified tissue culture method [25]. Fresh synovial tissues were minced and digested in 1 mg/mL collagenase (Sigma-Aldrich, MO, USA) for 3 h at 37°C to isolate synoviocytes. The cells were cultured with DMEM-Ham's F-12 (DMEM/F12) (Gibco, Shanghai, China), with 20% FCS (Gibco, Mulgrave, VIC, Australia) at 37°C and 5% CO_2_. FLS from passages three to five were used in this study.

### 2.3. Lentivirus and Stable Overexpression Cell Lines

Lentivirus containing empty plasmid or overexpression plasmid of TRIP was produced by using pLentif6/V5 (Life Technologies) vector, and lentivirus was obtained according to the manufacturer's instruction. The enriched lentivirus particle was used to infect FLS for 3 days at 50 MOI with the presence of polybrene; puromycin selection was performed to establish the overexpression cell lines.

### 2.4. RNA Extraction and Quantitative RT-PCR

Total RNA from FLS was extracted with TRIzol reagent according to the manufacturer's instructions (Invitrogen). Reverse transcription system (Takara, Dalian, China) was used to synthesize the complementary DNA. A LightCycler (ABI PRISM 7000; Applied Biosciences) and a SYBR RT-PCR kit (Takara Biotechnology) were used for quantitative RT-PCR analysis. Quantitative RT-PCR were performed using the following primer sequences: TRIP (sense), 5′-AGT GAA TTC ATC ATG CCT ATC CTC TCT CTG TG-3′; TRIP (antisense), 5′-CTG GGA TCC TCA CAT GTC TCG AAT CAT CTC CTC-3′; *β*-Actin (sense), 5′-ATG AA G ATC CTG ACC GAG CG-3′; *β*-Actin (antisense), 5′-TAC TTG CGC TGA GGA GGA GC-3′; IL-6 (sense), 5′-CTG CGC AGC TTT AAG GAG TTC-3′; IL-6 (antisense), 5′-CAA T CT GAG GTG CCC ATG CTA-3′; IL-1*β* (sense), 5′-CCA GCT ACG AAT CTC CGA CC-3′; IL-1*β* (antisense), 5′-CAT GGC CAC AAC AAC TGA CG-3′; TNF*α* (sense), 5′- GCT AAG AGG GAG AGA AGC AAC TAC A-3′; TNF*α* (antisense), 5′-GAA GAG GCT GA G GAA CAA GCA-3′; MMP-1 (sense), 5′-CCT GAA GAA TGA TGG GAG GCA-3′; MMP-1 (antisense), 5′-CTC TTG GCA AAT CTG GCG TG-3′; MMP-13 (sense), 5′-TCC TGG GCC AAA TTA TGG AG-3′; MMP-13 (antisense), 5′-TCC TGG GCC AAA TTA TGG AG-3′;

### 2.5. Immunoprecipitation and Western Blot Analysis

Collected RA-FLS extracts were lysed in immunoprecipitation buffer (Beyotime, Beijing, China); the supernatants were collected after centrifugation, followed by incubation with 1 *μ*g indicated antibody together with protein G Plus-Agarose Immunoprecipitation reagent (Santa Cruz Biotechnology, Inc.) for 5 hours. Beads were washed by immunoprecipitation buffer and immunoprecipitates were eluted by boiling. Before Western blot analysis, Bio-Rad quantification assay (Bio-Rad Laboratories, Hercules, CA) was used to measure lysates concentration. Proteins (25 *μ*g) were separated using 10% SDS-PAGE and transferred to a PVDF membrane (Millipore). The membrane was then blocked with 2.5% nonfat dry milk for 1 h. The antibodies for TRIP, TAK1, or ubiquitin (linkage-specific K48) (Abcam) and the antibodies specific for p65, phospho-p65, IKK*β*, phospho-IKK*β* (Cell Signaling Technology Inc, Beverly, MA), and *β*-Actin (Santa Cruz Biotechnology) were added and incubated overnight at 4°C. After incubation with the corresponding horseradish peroxidase-conjugated secondary antibody (Santa Cruz Biotechnology, CA, USA), the target protein was visualized by enhanced chemiluminescence (Thermo Fisher Scientific). Ubiquitination assay was performed as described [26], RIPA buffer contained 10 mM N-ethylmaleimide (Sigma), and 1% SDS was used to lyse cell. The cell lysates were denatured at 90°C for 5 min, followed by the dilution with SDS-free RIPA buffer until the concentration of SDS was decreased to 0.1%. Then immunoprecipitation assays were performed, and the samples were analyzed by western blot with indicated antibodies.

### 2.6. ELISA Assay

Protein levels of human IL-1*β* and IL-6 in cell-free FLS supernatants were measured using human IL-1*β* or IL-6 ELISA kit (R&D Systems, MN, USA) according to the manufacturer's instructions. Protein levels of human MMP-1 or MMP-13 in FLS supernatants were determined using Human MMP-1 or MMP-13 ELISA kit (R&D Systems, MN, USA).

### 2.7. Dual-Luciferase Reporter Gene Assays

The experiment was performed as described [23]. NF-*κ*B promoter region was cloned into pGL3-based vectors to construct Luciferase reporter plasmid. The reporter plasmid was transfected into RA-FLS-Ctrl or RA-FLS-TRIP groups using the Lipofectamine 2000 (Invitrogen, Shanghai, China), and phRL-TK plasmid was cotransfected as internal control. 36 hours later, the Luciferase activities were measured on a SpectraMax M5 reader (Molecular Devices, California, USA) using the Dual-Luciferase Reporter Assay System (Promega, Madison, USA).

### 2.8. Statistical Analysis

All data are expressed as mean ± SEM of three or four experiments. Statistical analysis was performed by two-tailed Student's *t*-test, with *p* < 0.05 being considered statistically significant.

## 3. Results

### 3.1. Expression of TRIP Was Downregulated in RA-FLS Compared with OA-FLS

**Figure 1:**
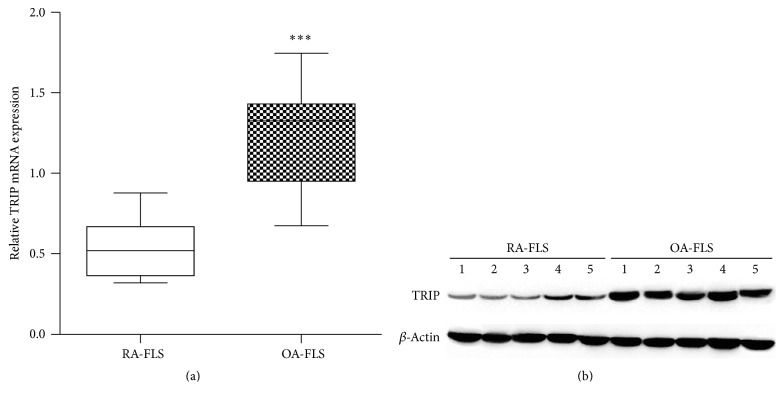
Expression of TRIP was downregulated in RA-FLS compared with OA-FLS. (a) qRT-PCR analysis of TRIP expression in FLS samples from 10 RA patients and 10 OA patients, quantitative analysis of TRIP expression was normalized to the expression of *β*-actin. (b) Western blot analysis of TRIP expression in FLS samples from 5 RA patients and 5 OA patients. ^*∗∗∗*^ *p* < 0.001.

To determine the role of TRIP in RA-FLS, we investigated the expression of TRIP in RA-FLS and OA-FLS at first. The mRNA expression of TRIP was quantified by quantitative reverse transcription-PCR (qRT-PCR) in FLS from 10 RA patients and 10 OA patients. As Figure 1(a) showed, mRNA level of TRIP was significantly attenuated in RA-FLS compared with OA-FLS. Furthermore, we confirmed that the expression level of TRIP protein was also significantly downregulated in RA-FLS compared with OA-FLS (Figure 1(b)).

### 3.2. Stable Overexpression of TRIP in RA-FLS by Using Lentivirus

**Figure 2:**
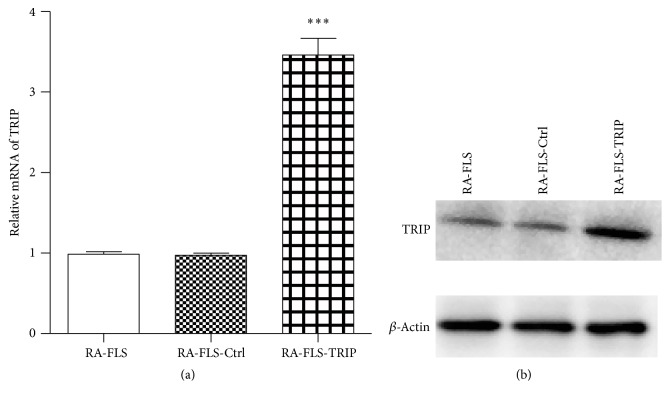
Stable overexpression of TRIP in RA-FLS. (a and b) Lentivirus carried TRIP overexpression plasmid or control plasmid were constructed and transfected into RA-FLS to build stable overexpression cell lines. The mRNA level (a) and protein level (b) of TRIP were measured. ^*∗∗∗*^ *p* < 0.001 versus RA-FLS-Ctrl group.

Because of the low expression of TRIP in RA-FLS, we used recombinant lentivirus containing overexpression plasmid of TRIP to investigated the function of TRIP in RA-FLS. The stable overexpression of TRIP in RA-FLS was analyzed. As shown in Figure 2(a), TRIP mRNA expression was significantly increased after transfection of recombinant lentivirus expressing TRIP. Consistent with the results of qRT-PCR, western blot analysis showed that TRIP protein expression was also increased by the infection of lentivirus contained TRIP overexpression plasmid compared to the control groups (Figure 2(b)). We named the cell line with TRIP stable overexpression for “RA-FLS-TRIP” and “RA-FLS-ctrl” for the cell line which was transfected with lentivirus contained empty vector and “RA-FLS” as a negative control not infected with lentivirus. These cell lines were used for the further studies.

### 3.3. Overexpression of TRIP Inhibited Inflammation in TNF*α*-Treated RA-FLS

**Figure 3:**
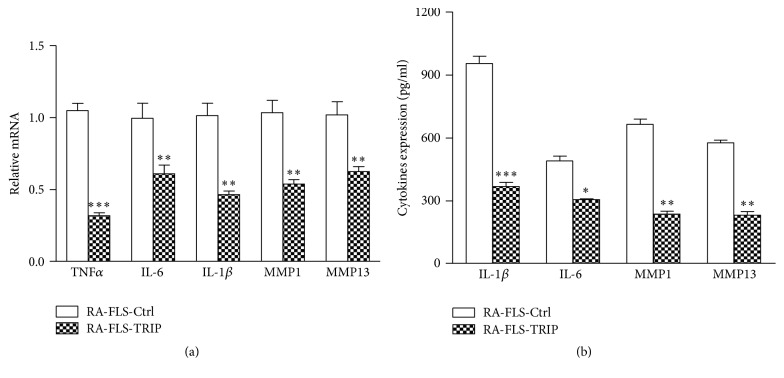
Overexpression of TRIP inhibited inflammation in TNF*α*-treated RA-FLS. (a) qRT-PCR analysis of the mRNA level of inflammatory cytokines and MMPs in RA-FLS-Ctrl or RA-FLS-TRIP group exposed to TNF*α* (100 ng/mL) stimulation for 60 min. (b) ELISA analysis of the secreted protein level of inflammatory cytokines and MMPs in RA-FLS-Ctrl or RA-FLS-TRIP group exposed to TNF*α* (100 ng/mL) stimulation for 60 min. ^*∗*^ *p* < 0.05, ^*∗∗*^ *p* < 0.01, and ^*∗∗∗*^ *p* < 0.001 versus respective control group.

TRIP was reported to inhibit TNF*α*-induced NF-*κ*B activation [27], and TNF*α* is involved in RA pathogenesis, so we evaluated the effects of TRIP overexpression on the production of proinflammatory cytokines and MMPs in TNF*α*-treated RA-FLS to investigate the potential role of TRIP in regulating inflammatory process in RA-FLS. TRIP overexpression significantly suppressed the mRNA expression of TNF*α*, IL-6, IL-1*β*, MMP1, and MMP13 in TNF*α*-stimulated RA-FLS (Figure 3(a)). Furthermore, we examined the protein level of these inflammatory mediators in supernatants by ELISA, and the secreted protein level of IL-6, IL-1*β*, MMP1, and MMP13 was also suppressed in TRIP overexpressed RA-FLS compared with RA-FLS-Ctrl group after TNF*α* treated (Figure 3(b)). These data indicated that TRIP overexpression could inhibit the production of inflammatory cytokines and MMPs in TNF*α*-treated RA-FLS.

### 3.4. Overexpression of TRIP Suppressed TNF*α*-Induced NF-*κ*B Activation in RA-FLS

**Figure 4:**
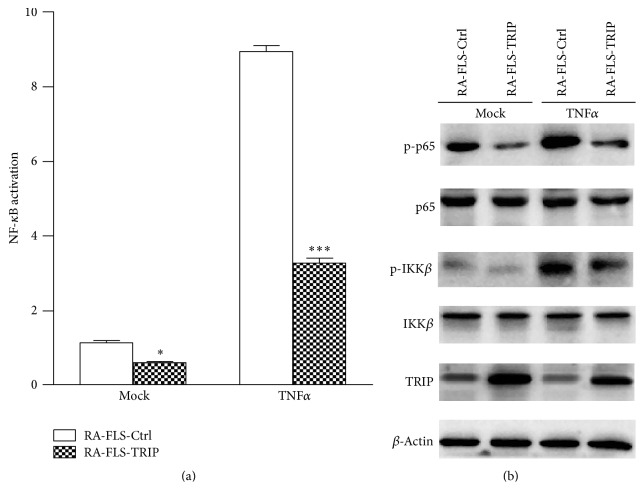
Overexpression of TRIP suppressed TNF*α*-induced NF-*κ*B activation in RA-FLS. (a) RA-FLS-Ctrl and RA-FLS-TRIP cell lines were transfected with NF-*κ*B reporter plasmid together with phRL-TK plasmid (internal control), 36 hours later the cells were stimulated with TNF*α* for 60 min, and the activation of NF-*κ*B promoter was measured by Dual-Luciferase reporter gene assay. (b) Western blot analysis of the phosphorylation of p65 and IKK*β* in RA-FLS-Ctrl or RA-FLS-TRIP group exposed to TNF*α* (100 ng/mL) stimulation for 60 min. ^*∗*^ *p* < 0.05, ^*∗∗∗*^ *p* < 0.001 versus respective control group.

Production of proinflammatory cytokines upon TNF*α* depends mainly on the NF-*κ*B activation, so we hypothesized if TRIP would affect NF-*κ*B activation in RA-FLS. We used Dual-Luciferase Reporter Assay to examine the activation of NF-*κ*B signaling, and we found that the promoter activation of NF-*κ*B was significantly suppressed in RA-FLS-TRIP group compared with control group (Figure 4(a)). TRIP overexpression also suppressed the phosphorylation of p65 and IKK*β*, indicating the inhibition of NF-*κ*B pathway (Figure 4(b)). Interestingly, we also found that, without TNF*α* stimulated, TRIP would inhibit NF-*κ*B activation, but after TNF*α* is added into culture medium, the inhibitory effect of TRIP was significantly elevated.

### 3.5. TRIP Interacted with TAK1 and Promoted TAK1 Degradation in RA-FLS

**Figure 5:**
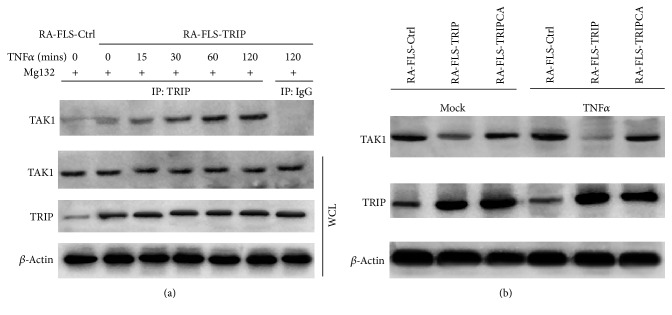
TRIP interacted with TAK1 and promoted TAK1 degradation in RA-FLS. (a) Extracts of RA-FLS stimulated with TNF*α* (100 ng/mL) for various times were subjected to immunoprecipitation with anti-TRIP or control anti-IgG and immunoblot analysis with individual antibodies. Mg132, proteasome inhibitor. WCL, whole cell lysates (b) Western blot analysis of the expression of TAK1 in RA-FLS cells stably expressed control or TRIP (wild type) or TRIPCA (E3 ligase activity mutation) plasmid stimulated with TNF*α* (100 ng/mL) for 120 min.

TRIP has been identified to repress TLR3/4 and RIG-I signaling by interacting with TBK1 and promoting TBK1 degradation through its E3 ligase activity [17]. We assumed whether TRIP could affect NF-*κ*B activation through its RING domain in RA-FLS. TAK1 are the most abundant in inflamed synovium as well as cultured FLS [28] and the function of TAK1 in NF-*κ*B singling is similar to TBK1 in TLR pathways, so we examined whether TRIP could regulate TAK1 activity. Interestingly, we found that TRIP could interact with TAK1 in unstimulated RA-FLS-Ctrl group, whereas the interaction was significantly improved in TRIP overexpressed RA-FLS after TNF*α* is added and the interaction was time dependent in the presence of proteasome inhibitor Mg132 (Figure 5(a)). As a control, the interaction could not be detected with normal IgG.

Furthermore, we assumed if the abundant expression of TAK1 is related to the low expression of TRIP in RA-FLS. We investigated the expression of TAK1 in RA-FLS cells stably expressed control or TRIP (wild type) or TRIPCA (E3 ligase activity mutation) plasmid in the absence or presence of TNF*α*; the results showed that TAK1 expression was decreased in RA-FLS-TRIP group compared with control group without TNF*α* stimulated, whereas with the TNF*α* added into the culture medium, the expression of TAK1 significantly decreased in TRIP stable transfection RA-FLS compared with control RA-FLS (Figure 5(b)). At last, we found that overexpression of TRIPCA which was a mutant plasmid lost E3 ligase activity of TRIP could not promote TAK1 degradation (Figure 5(b)). Taken together, we found that TRIP interacted with TAK1 and promoted TAK1 degradation dependent on its E3 ligase activity.

### 3.6. TRIP Promoted K48-Linked Ubiquitination of TAK1 in RA-FLS

**Figure 6:**
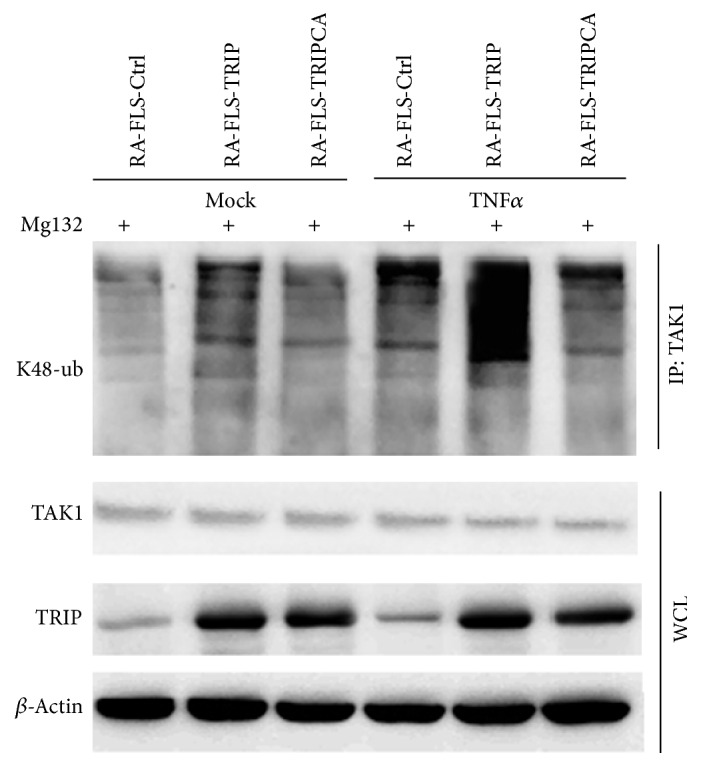
TRIP promoted K48-linked polyubiquitination of TAK1 in RA-FLS. Extracts of control RA-FLS and RA-FLS stably overexpressed TRIP or TRIPCA stimulated with TNF*α* (100 ng/mL) for 120 min were subjected to immunoprecipitation with anti-TAK1 and immunoblot analysis with antibody specific for K48-linked ubiquitin. Mg132, proteasome inhibitor. WCL, whole cell lysates.

We certified that the E3 ligase activity is crucial for TRIP mediated TAK1 degradation, and the K48-linked protein ubiquitination leads to the degradation of the corresponding protein by 26S proteasome; therefore, we examined whether TRIP could affect the K48-linked ubiquitination of TAK1. After Mg132 treatment, the K48 linked ubiquitination level of TAK1 was increased in RA-FLS-TRIP group compared with control group (Figure 6), and TRIP mediated K48-linked ubiquitination level of TAK1 was increased significantly when TNF*α* existed. Importantly, the RA-FLS stably overexpressed TRIP mutation plasmid (TRIPCA) lost the ability to promote polyubiquitination of TAK1 (Figure 6), indicating TRIP could promote the K48-linked ubiquitination of TAK1 through the RING-finger domain.

## 4. Discussion

In the current study, we demonstrated the expression and function of TRIP in the human RA-FLS. To the best of our knowledge, this study is the first publication showing the relationship between TRIP and RA.

The cell number of FLS increases significantly when the constant inflammatory processes induced by inflammatory cytokines happen in the synovial tissue [29]. These FLS cells contribute to the inflammatory microenvironment and recruit and activate more immune cells to the damaged area by secreting various proinflammatory cytokines and chemokines, especially IL-6, IL-8, and MMPs, and thereby contribute to cartilage damage and joint destruction [30, 31].

TNF*α* is one of the major mediators involved in RA. Stimulation of TNF*α* results in the proliferation of FLS and increased production of inflammatory cytokines and enzymes. As a positive feedback, FLS and immune cells activated by TNF*α* could also lead to the continuous production of cytokines mainly through NF-*κ*B signaling pathway, leading to the aggravation of synovial inflammation and joint destruction [32–35]. Thus, it might be an attractive strategy for the treatment of RA to target intracellular pathways such as NF-*κ*B signaling [23, 36–38].

TRIP is a member of the RING-type E3 ubiquitin ligase family that undergoes autoubiquitination but its E3 ubiquitin ligase substrates need further study [15]. Almeida et al. postulated that TRIP may promote the polyubiquitination and proteasome dependent degradation of cell cycle regulatory proteins [19]. Recently, TRIP was found to directly interact with TANK-binding kinase 1 (TBK1) and promote TBK1 K48-linked ubiquitination and degradation, therefore negatively regulating IFN-*β* production and antiviral response [15]. Historically, TRIP was reported to interact with the tumor necrosis factor (TNF) receptor-associated factors (TRAFs) and ectopic expression of TRIP repressed NF-*κ*B signaling in a RING domain independent manner [15, 27]. Although the function of TRIP that inhibit TNF*α*-induced NF-*κ*B activation had been certified in breast epithelial cells [18], whether there was E3 ligase substrate of TRIP in NF-*κ*B signaling still remains unknown. Similar to their researches, we found TRIP inhibit TNF*α*-induced NF-*κ*B pathway activation in RA, and we revealed the potential E3 ligase substrate target of TRIP in NF-*κ*B signaling in our current study.

TAK1 is one of the most crucial regulators of the innate immunity and the proinflammatory signaling pathway. It mediates the activation of diverse pathways including NF-*κ*B, c-Jun N-terminal kinase (JNK), and p38 pathways in response to TNF*α*, IL-1, and toll-like receptor agonists. It has been reported that K63-linked polyubiquitination of TAK1 is necessary for NF-*κ*B activation [39–44], and the K48-linked TAK1 polyubiquitination could lead to the degradation of TAK1 and termination TNF*α*-induced NF-*κ*B activation [45, 46]. Recently, ITCH was reported to play as an E3 ligase of TAK1 to promote K48-linked polyubiquitination of TAK1 and suppressed TNF*α*-induced NF-*κ*B activation [46]. Similar to their results, in this current research, we found that TRIP can mediate TAK1 K48-linked polyubiquitination and promote TAK1 degradation, leading to the inhibition of TNF*α*-induced NF-*κ*B activation in RA-FLS. Taken together with our result that TRIP expression was downregulated in RA-FLS compared with OA-FLS, it would help us understand previous reported research that abundant expression of TAK1 was observed in inflamed synovium and cultured FLS [28].

It seemed that TRIP could interact with TAK1 and promote TAK1 K48-linked polyubiquitination and degradation without stimuli added, it may be caused by the self-production of TNF*α* or other cytokines of RA-FLS, and the weak interaction between TRIP and TAK1 may be due to the low level of these cytokines of unstimulated RA-FLS. Whereas the most worthy of our attention is that after TNF*α* stimulated, the interaction between TRIP and TAK1 was significantly enhanced, and the ubiquitination level of TAK1 was obviously increased, together with the marked decreased of TAK1 expression in RA-FLS.

In conclusion, our current study provided the evidence that TRIP expression was attenuated in RA-FLS compared with OA-FLS, and overexpression of TRIP significantly inhibited the activation of NF-*κ*B signaling and decreased the production of proinflammatory cytokines and MMPs in TNF*α*-stimulated RA-FLS. Furthermore, we found that TRIP could interact with TAK1 and promoted K48-linked polyubiquitination and degradation of TAK1 in RA-FLS. These results suggest that TRIP plays an anti-inflammatory role in RA, and TRIP is a potential target for treatment of RA.
